# Zebras Masquerading As Horses: A Case of Spinal Subarachnoid Haemorrhage

**DOI:** 10.7759/cureus.19511

**Published:** 2021-11-12

**Authors:** Zeshan K Saeed, Kerry Fisher, Sunil Melath, Bushra Omar

**Affiliations:** 1 Rheumatology, Frimley Park Hospital, Frimley, GBR; 2 Rheumatology, Royal Berkshire Hospital, Reading, GBR; 3 General Practice, Frimley Park Hospital, Frimley, GBR

**Keywords:** upper motor neurone, haemorrhagic csf, arteriovenous malformation, cervical subarachnoid haemorrhage, spinal subarachnoid haemorrhage

## Abstract

A previously independent 83-year-old lady presents with acute confusion, decreased mobility, urinary retention, and constipation, having recently received a course of oral acyclovir for shingles. The patient was noted to have extensive bruising to her upper limbs, and blood tests showed raised inflammatory markers with low platelet count, although this remained above 75 × 10^9^/L. Her confusion on a background of shingles raised the differential diagnosis of varicella-zoster virus (VZV) encephalitis. CT head and MRI brain showed no acute intracranial abnormality. Lumbar puncture yielded frankly haemorrhagic cerebrospinal fluid (CSF), but viral polymerase chain reaction (PCR) testing was negative for the varicella-zoster virus. She later developed further right shoulder pain and right lower limb weakness three days post-initial lumbar puncture. Repeat CT head was unremarkable. MRI spine showed extensive spinal subarachnoid haemorrhage, with possible cervical arteriovenous malformation and L5/S1 spinal nerve compression. The patient was managed conservatively with dexamethasone and inpatient physiotherapy support. She was discharged after a long hospital stay at a new mobility baseline requiring hoist transfers.

## Introduction

Subarachnoid haemorrhage (SAH) represents a cause of CNS bleeding, affecting 5.6 per 100,000 patients worldwide per year [1]. Spinal arachnoid haemorrhage is a rare cause of SAH representing <0.1% of all SAH and is commonly related to trauma, vascular lesions, neoplasms, or coagulopathy [2]. Patients often present with non-specific neurological symptoms including lumbago, headache, and paraparesis [3], and may also present similarly to those with cerebral haemorrhages [4]. Whilst traditional investigations may include cranial imaging, due to its rarity and non-specific presentation, spinal imaging may not be considered. This leads to diagnostic delays, with time to diagnosis ranging from one day to two months [3]. We report a case of spinal subarachnoid haemorrhage in an 83-year-old female, which highlights the challenges of diagnosis and raises awareness for the condition.

## Case presentation

A previously independent 83-year-old lady presented with a 10-day history of acute confusion, decreased mobility, urinary retention, and constipation. Significant past medical history included ischaemic stroke, atrial fibrillation (AF) on apixaban, polymyalgia rheumatic with bilateral shoulder pain, and obstructive sleep apnoea. The patient was receiving oral acyclovir prior to admission for shingles affecting the genital area. On examination the patient was hypertensive 202/102 mmHg. Extensive bruising was noted over her forearms attributed to anticoagulation. Several tenders, crusted lesions were noted on the labia, with smaller lesions affecting the buttocks and perineum attributed to shingles. Her family reported these were improving with acyclovir. Her cardiorespiratory examination was normal, her abdomen was distended, with urinary retention of 900 mls. Neurological examination showed normal tone, power, sensation and reflexes in all four limbs, and no cranial nerve abnormalities.

Investigations

**Figure 1:**
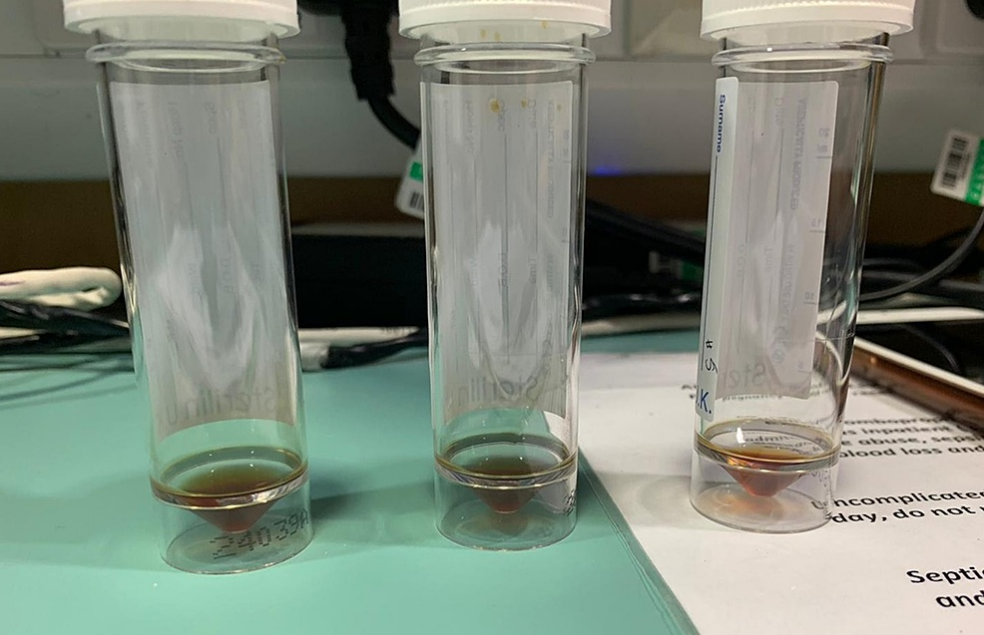
Image of CSF from first LP. Image of haemorrhagic CSF. CSF: cerebrospinal fluid. LP: lumbar puncture.

Blood tests revealed hyponatraemia (128 mmol/L), hypokalaemia (3.2 mmol/L), C-reactive protein (CRP) (23 mg/L), and low platelet count (88 × 10^9^/L). Paired serum and plasma osmolalities were indicative of Syndrome of Inappropriate Antidiuretic Hormone Secretion (SIADH). Initial CT head showed no acute intracranial phenomena. Genital swab confirmed varicella-zoster virus (VZV) infection. Her confusion was initially felt to be multifactorial due to hyponatraemia, constipation, and urinary retention. The patient was catheterised and placed on fluid restriction, anticoagulation was held whilst monitoring platelet count, and given her raised inflammatory markers and genital swab positive for VZV, she was commenced on intravenous acyclovir. Sodium levels improved, however, platelet count and haemoglobin levels declined with no obvious source of bleeding. She exhibited fluctuating cognition and later developed right leg weakness and brisk deep tendon reflexes, which raised the possibility of VZV encephalitis. Lumbar puncture (LP) revealed frankly haemorrhagic cerebrospinal fluid (CSF) (Figure 1). 

Repeat CT head showed no evidence of intracranial haemorrhage. MRI brain was normal. The patient was commenced on ceftriaxone in addition to acyclovir, whilst awaiting CSF culture results. CSF and radiological findings were discussed with the local tertiary neurosurgical centre, who advised repeat lumbar puncture to exclude a traumatic tap. This again yielded haemorrhagic CSF. Samples were negative for any viral or bacterial pathogens (Table 1), and ceftriaxone and acyclovir stopped after 14 days. 

**Table 1:** Lumbar puncture results. PCR: polymerase chain reaction, TB: tuberculosis.

	Lumbar puncture 1	Lumbar puncture 2
Red blood cells: first sample (×10^6^/L)	177,720	15,600
Red blood cells: last sample (×10^6^/L)	14,080	14,600
White blood cells (×10^6^/L)	52	316
Polymorphs (%)	19	80
Lymphocytes (%)	81	20
Organisms	None seen	None seen
Culture report	No growth	No growth
Viral PCR (herpes simplex virus, varicella-zoster virus, enterovirus, parvovirus)	-	Negative
Meningococcal/pneumococcal PCR	-	Negative
TB PCR	-	Negative
Glucose (mmol/L)	0.7	2.5

**Figure 2:**
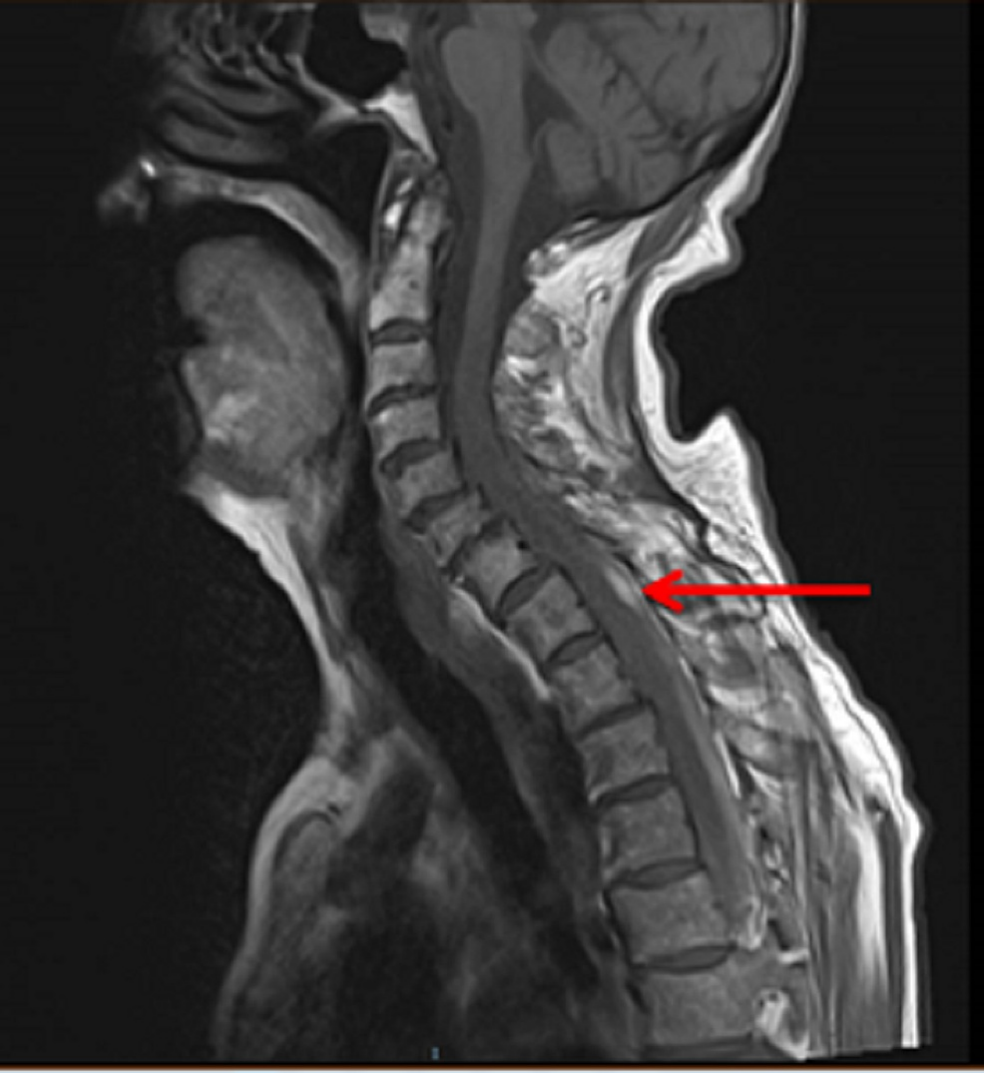
Sagittal T1-weighted sequence cervical and upper thoracic spine. Normal CSF is of low signal intensity and this is seen within the spinal canal at the cervical spine level. The spinal subarachnoid haemorrhage is demonstrated as abnormal high signal intensity posterior to the cord at the level of the T1 vertebral body (red arrow), this extends caudally all the way down the spinal canal and is seen within the distal thoracic and lumbar spine. CSF: cerebrospinal fluid.

**Figure 3:**
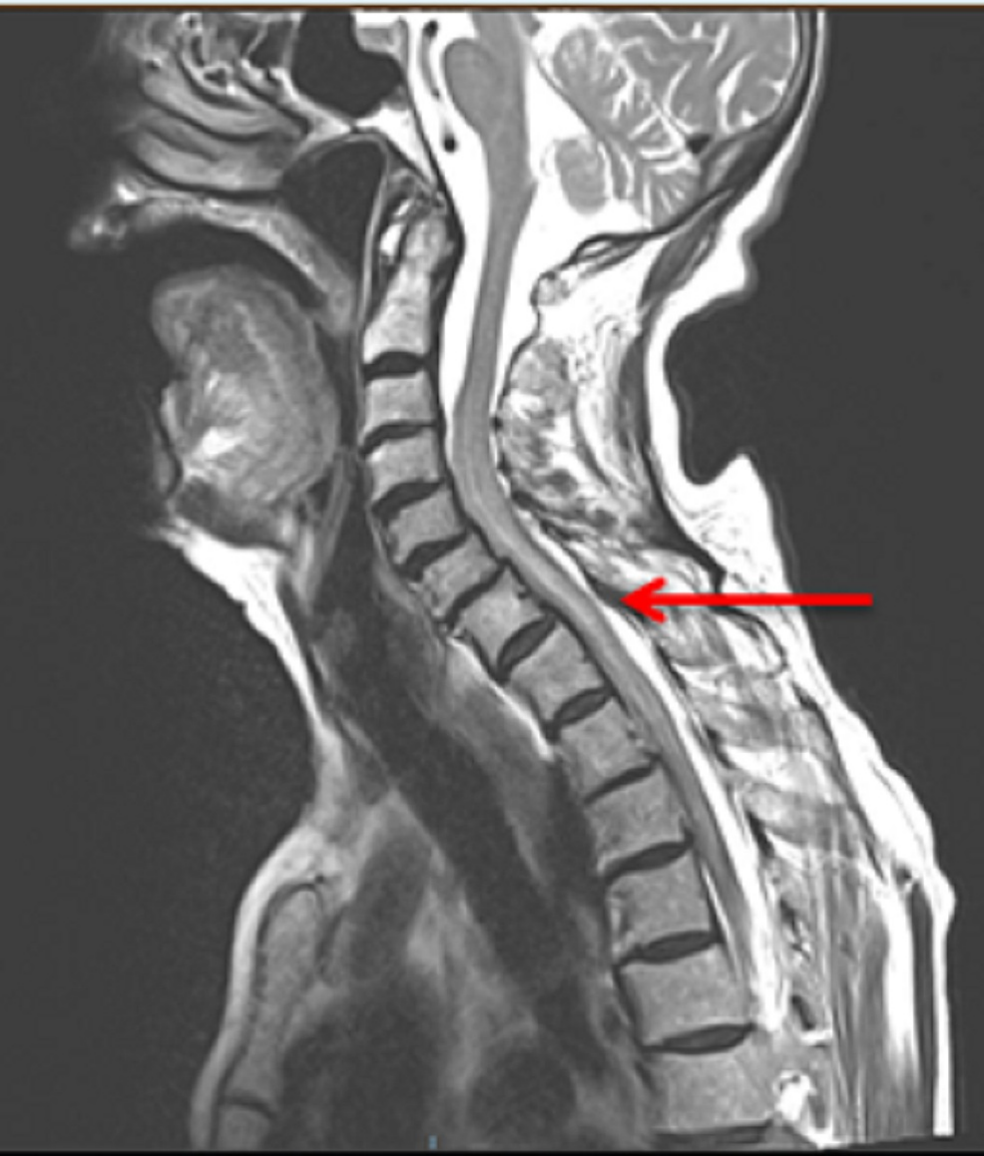
Sagittal T2-weighted sequence cervical and upper thoracic spine. The spinal subarachnoid haemorrhage is demonstrated as abnormal low signal intensity posterior to the T1 vertebral body (red arrow). It is important to view the T1 and T2 images together. Blood can be of varying signal intensity depending on the acuity of the haemorrhage as different stages of blood products have varying paramagnetic qualities.

**Figure 4:**
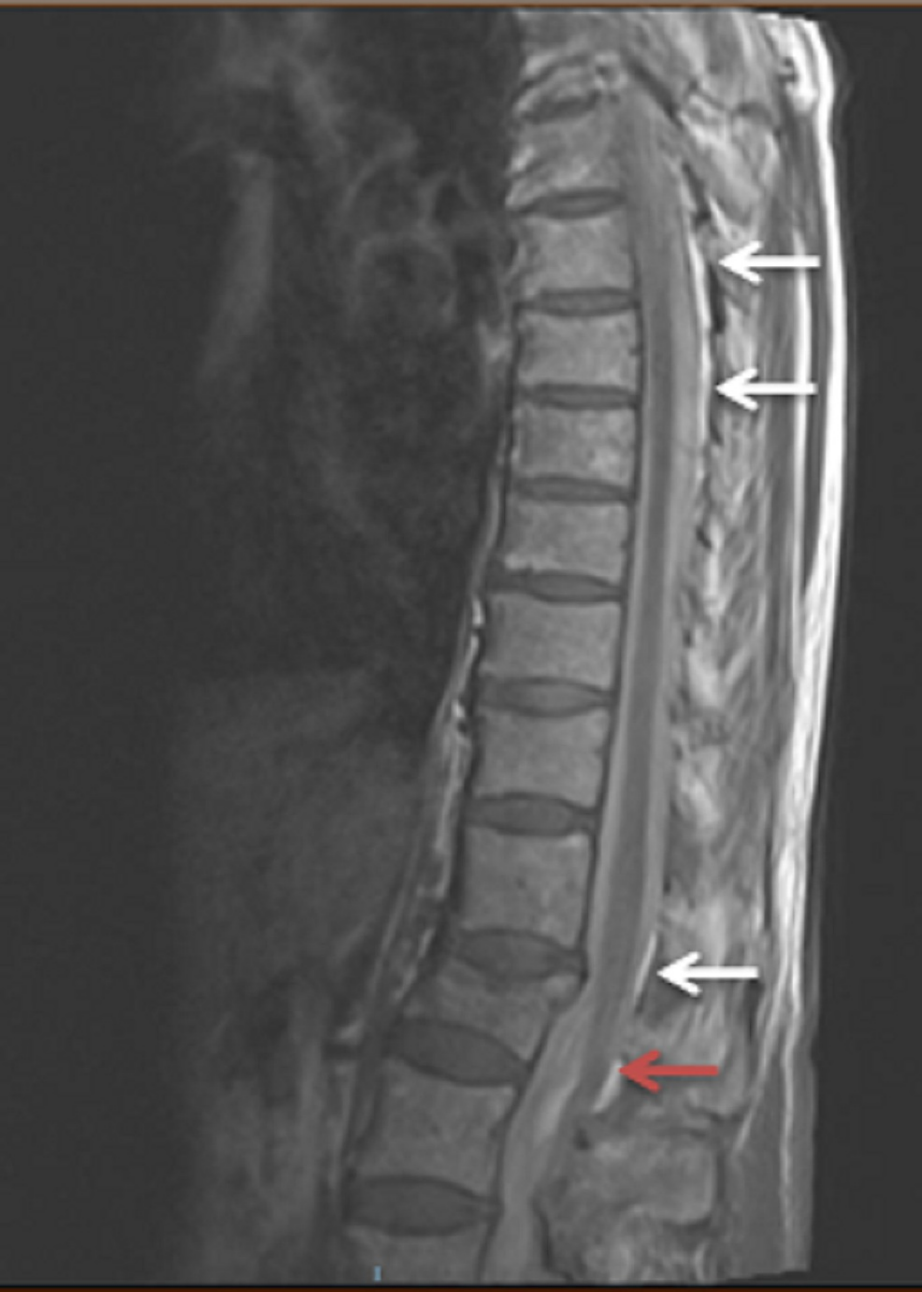
Sagittal T1-weighted sequence lumbar spine. The spinal subarachnoid haemorrhage is demonstrated as abnormal high signal intensity posterior to the cord extending distally within the thoracic (white arrows) and lumbar spine (red arrow)

**Figure 5:**
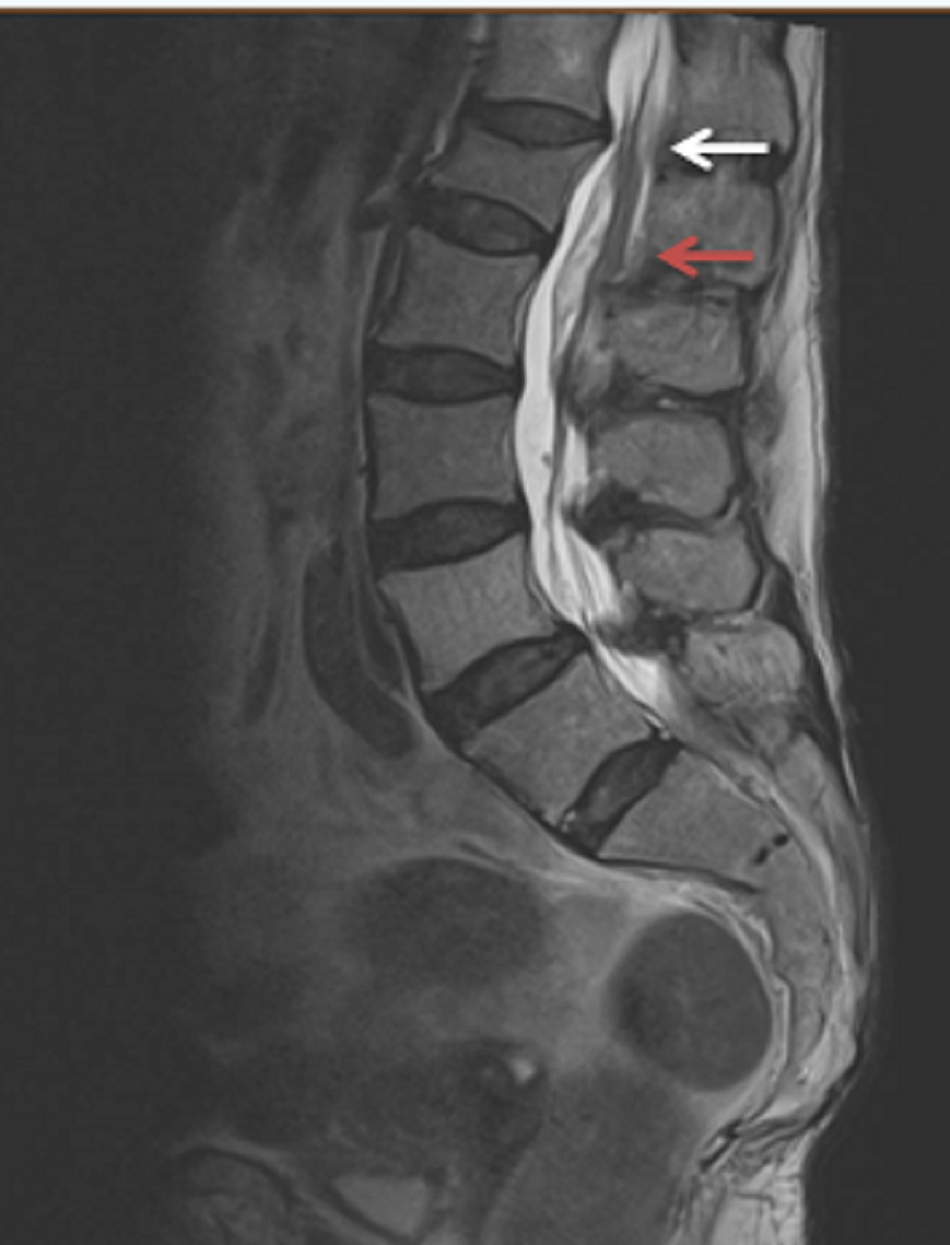
Sagittal T2-weighted sequence lumbar spine. The spinal subarachnoid haemorrhage is demonstrated as abnormal hypo to isointense signal intensity posterior to the cord at the thoracolumbar junction (white arrow) and lumbar region (red arrow) with associated narrowing.

The patient exhibited progressive neurological signs in the right lower limb, with increased tone, weakness and upgoing plantar response, and noted new right shoulder pain. She underwent multiple reviews by neurology and discussion with microbiology and the initial concern following her lumbar puncture was regarding possible tuberculosis meningitis or possible, varicella-zoster virus (VZV) encephalitis resulting in bleeding or VZV vasculitis. Further review by the neurology team, who advised a CT aortic angiogram to assess for possible spinal intrathecal haemorrhage. This was reviewed in a neuroradiology meeting and showed blood within the subdural and epidural space of the thoracic spine. MRI spine showed normal CSF appearances above the C4 vertebra (Figures 2, 3) but extensive extra-axial haemorrhage below this extending to the sacrum (Figures 4, 5), with a focal point of high intensity at T1 (Figure 2). There was a serpinginous lesion within the lower cervical cord exiting to the right side, suggestive of a lower cervical arteriovenous malformation (AVM) (Figure 3). There was also cord oedema with lateral recess narrowing at L4/L5 and L5/S1 with compression of the traversing L5 nerve roots and right S1 nerve roots, in keeping with her lower limb neurology and urinary retention (Figure 5).

Treatment

The patient was transferred to a tertiary neurosurgical centre. Repeat MRI spine and magnetic resonance angiography (MRA) with contrast showed a fixed subarachnoid haematoma throughout the spine, but no abnormal vasculature. These findings remained in keeping with an AVM, but at multidisciplinary team discussion, it was felt that neurosurgical intervention would be inappropriate due to extent of spinal cord lesion and oedema present. The patient was repatriated for conservative management with dexamethasone for spinal cord oedema, physiotherapy, and occupational therapy. 

Outcome and follow-up

After repatriation, this patient had a long hospital stay whilst receiving physiotherapy and occupational therapy. However, it was not felt that she would return to her pre-admission mobility. At discharge, she continued to have fluctuating cognition, with right lower limb weakness due to spinal pathology and left lower limb weakness due to lack of use. She was discharged to her own home requiring hoisted transfers and a package of care, with a plan for repeat imaging and follow-up at the tertiary neurosurgical centre. This patient died three months after discharge, and it is unclear whether repeat imaging took place.

## Discussion

Spinal SAH represents <1% of all subarachnoid bleeds and carries a high risk of permanent neurological deficits and mortality [4] as seen in this case. Due to the low prevalence, lack of clinical awareness and non-specific presenting symptoms, there are often delays in diagnosis. This is clear in this case, where the time to diagnosis was 25 days. This was despite a review by neuroradiology and neurology. On discussion within our general medical team looking after the patient and further repeat lumbar puncture by another independent consultant within our district general hospital indeed the case presentation and location as a spinal arachnoid haemorrhage was not known about. Indeed on discussion with the local world-renowned neurosurgical centre, the advice was to repeat LP to exclude a traumatic tap. Multiple neurology reviews by experienced consultants felt that the findings were in potential keeping with initially TB meningitis or possible VZV vasculitis or encephalitis causing a spinal bleed. The patient having a positive swab for VZV on her genital ulcers, although CSF PCR for VZV was negative added additional difficulty in establishing the correct diagnosis. Initial computed tomography angiography (CTA) was undertaken 10 days post the initial lumbar puncture, and a small possible aneurysm was only identified following discussion in neuroradiology multi-disciplinary team (MDT). MRI spine was undertaken 14 days post-initial lumbar puncture and four days post this due to concerns with the patient's Glasgow Coma Scale (GCS) and ensuring she was able to tolerate an MRI scan. These all lead to a significant delay in diagnosis.

The mechanism of bleeding is thought to be related to episodes of minimal exertion which increase intrathoracic pressure, and consequently intraluminal pressures of subarachnoid spinal vessels, can increase the risk of spinal vessel rupture [5]. Key risk factors reflect those of cerebral strokes: family history, hypertension, smoking, alcohol intake >2 units/day, vascular malformations, and collagen disorders [1].

In this patient with likely underlying AVM and a history of obstructive sleep apnoea, changes in intrathoracic pressure may have caused the rupture of AVM vessels. Haemorrhage would then be accelerated due to anticoagulation and low platelet counts. Patients present similarly to cerebral haemorrhage [4], but more specifically note pain between the shoulder blades or along the spine as seen in this patient with right shoulder pain. This is followed by features of cord compression signalling the level of spinal involvement, haematoma extension, and cord oedema [1,4].

MRI and MRA remain the first-line diagnostic imaging techniques [1], with most haemorrhages occurring dorsally in the cervicothoracic or lumbar regions. Ventral haemorrhages are noted to cause fewer neurological deficits and are therefore less frequently investigated [2]. Haematoma formation within the spine is impaired by dilution by CSF and spinal cord pulsation [2]. This leads to prolonged bleeding time and haematomas involving the entire spine even in patients with normal clotting function. Patients may require surgical spinal decompression; however, this was not needed in this case.

Given the confounding additional factors, there was a delay in diagnosis. The rarity of the condition means that many experienced doctors are unaware of the condition, and we feel it is important to raise awareness of the condition. We would propose that for patients with CSF that is entirely consistent with large volume haemorrhage with a normal CT head/MRI head that there should be early scanning of the spinal cord with an MRI/MRA to look for signs of haemorrhage and aneurysm. These imaging techniques are now widely available and easily accessible. 

## Conclusions

Although rare, spinal subarachnoid haemorrhage should be considered as a cause of CNS bleed in a patient with progressive upper motor neurone signs, neck or shoulder pain, and haemorrhagic CSF. Neurological features often indicate the level of spinal cord involvement for focussed spinal imaging. Greater awareness of this condition is warranted amongst clinicians in an effort to reduce diagnostic delay, morbidity, and mortality. If CSF shows evidence of a haemorrhage urgent spinal imaging for the present of haemorrhage and for aneurysm is warranted and necessary.
